# Pandemic paradox: traffic mortality trends during COVID-19 in Campinas, 2019-2023

**DOI:** 10.1590/1980-549720250050

**Published:** 2025-11-03

**Authors:** Vitor Favali Kruger, Thiago Rodrigues Araujo Calderan, Elcio Shiyoiti Hirano, Gustavo Pereira Fraga

**Affiliations:** I Universidade de Campinas, School of Medical Sciences, Department of Surgery, Division of Trauma Surgery - Campinas (SP), Brazil.

**Keywords:** COVID-19, Traffic accidents, Mortality, Accident prevention, Wounds and injuries, Accident, traffic, COVID-19, Acidentes de trânsito, Mortalidade, Prevenção de acidentes, Ferimentos e lesões

## Abstract

**Objective:**

The aim of this study was to analyze road traffic mortality patterns during pre-pandemic, pandemic, and post-pandemic periods in Campinas, Brazil.

**Methods:**

This is a retrospective observational study conducted in Campinas from 2019 to 2023, analyzing 17,726 road traffic crashes with 406 deaths, using databases from the Campinas Municipal Development Company and São Paulo State Traffic Accident Information System. Multivariate logistic regression analysis was performed to identify independent risk factors associated with road traffic deaths. Risk factors analyzed included alcohol consumption, nighttime driving, gender, crash location, vehicle type, and weekend occurrence. Chi-square tests were used to compare proportions across periods.

**Results:**

Despite a 32.0% reduction in traffic volume, mortality rates increased from 10.46 to 13.76 per 100,000 inhabitants, with a 26.7% increase in years of potential life lost. The frequency of road traffic deaths increased from one death every 2.9 days to one every 2.0 days. Speeding was the main contributing factor for violations, representing 67% during the pandemic period. The highest risk emerged from the combination of alcohol consumption, speeding, and nighttime driving. Motorcyclists accounted for 43.1% of deaths, increasing to 47.0% in the post-pandemic period.

**Conclusion:**

A pandemic paradox emerged where reduced traffic led to increased mortality. Risk behaviors established during the pandemic became entrenched rather than temporary, particularly affecting young male motorcyclists.

## RESUMO

**Objetivo:**

Analisar os padrões de mortalidade no trânsito durante os períodos pré-pandêmico, pandêmico e pós-pandêmico em Campinas, Brasil.

**Métodos:**

Estudo observacional retrospectivo realizado em Campinas entre 2019 e 2023, analisando 17.726 sinistros de trânsito, com 406 óbitos, utilizando bancos de dados da Empresa Municipal de Desenvolvimento de Campinas e Sistema de Informações de Acidentes de Trânsito do Estado de São Paulo. Foi realizada análise de regressão logística multivariada para identificar fatores de risco independentes associados aos óbitos decorrentes de sinistros de trânsito, como alcoolemia, período noturno, gênero, localização da via, tipo de veículo e final de semana. Testes ꭓ^2^ foram utilizados para comparar proporções entre os períodos.

**Resultados:**

Apesar da redução de 32,0% no volume de tráfego, as taxas de mortalidade aumentaram de 10,46 para 13,76/100.000 habitantes, com elevação de 26,7% nos anos potenciais de vida perdidos. A frequência de colisões com vítimas fatais aumentou de uma morte a cada 2,9 dias para uma a cada 2,0 dias. O excesso de velocidade constituiu o principal fator contribuinte das infrações, representando 67% no período de pandemia. Os maiores risco emergiram da combinação entre consumo de álcool com direção noturna e excesso de velocidade. Motociclistas representaram 43,1% dos óbitos, aumentando para 47,0% no período pós-pandêmico.

**Conclusão:**

Emergiu um paradoxo pandêmico onde a redução do tráfego conduziu ao aumento da mortalidade. Comportamentos de risco estabelecidos durante a pandemia tornaram-se permanentes ao invés de temporários, afetando particularmente motociclistas jovens do sexo masculino.

## INTRODUCTION

The Global status report on road safety 2023^1^ of the World Health Organization estimated 1.19 million road traffic deaths in 2021, corresponding to 15 deaths per 100,000 inhabitants. Road traffic injuries (RTIs) are the leading cause of death among those aged 5-29 years, with 69.0% of all fatalities occurring during the productive years (18-59 years). The socioeconomic burden of RTIs extends beyond immediate casualties. The human capital approach aggregates individual costs such as healthcare costs and societal impacts due to loss of labor productivity and long-term disability, with an annual economic impact estimated at 1.0-3.0% of the gross domestic product, reaching 6.0% in some cases. This impact reverberates both on the social security system, overloading social protection, and on the weakening of the family economic structure, creating cycles of socioeconomic vulnerability. A study by Viana et al.^2^, conducted from 2012 to 2022, complements this econometric analysis, demonstrating that the Brazilian public health system allocated US$38 million for the care of approximately 1.6 million road traffic crash victims during the analyzed period, with an average cost per hospitalization of US$239.66. Gender disparity is notable, with men disproportionately affected at a female-to-male fatality ratio of 1/3, particularly work-related fatalities.

In the global road safety landscape, powered two- and three-wheeler users accounted for 30.0% of road traffic deaths, four-wheel vehicle occupants for 25.0%, and pedestrians for 21.0%, with marked variations between high-income countries and low- and middle-income countries. This disparity is particularly relevant in Brazil, where motorcycle-related deaths represent a critical public health concern, especially in metropolitan areas^3^.

The COVID-19 pandemic, declared by the WHO on March 11, 2020, exacerbated existing healthcare challenges and led to significant changes in mobility patterns through containment measures. Despite reduced traffic volumes, some regions reported increased RTI severity, indicating complex interactions between mobility restrictions and driver behavior. Campinas offers a unique opportunity to examine these effects due to its comprehensive trauma system and diverse road network that combines urban and highway traffic patterns. The objective of this study was to analyze changes in road traffic death patterns, crash severity, and risk factors across three periods (pre-pandemic, pandemic, and post-pandemic). We examined the impact of mobility restrictions on traffic safety outcomes, behavioral adaptations, and their effects on different road user groups to inform evidence-based traffic safety policies and intervention strategies.

## METHODS

This retrospective observational study analyzed RTIs in Campinas, Brazil, across three distinct 1-year periods: pre-pandemic (January 2019-January 2020), pandemic (April 2020-April 2021), and post-pandemic (January 2022-January 2023). The pandemic period encompassed Brazil’s first wave (March-September 2020) and second wave (November 2020-March 2021), which included two government-mandated lockdowns in Campinas: March 21-May 10, 2020 (50 days), and March 15-April 11, 2021 (27 days), during the Emergency Phase of the São Paulo Plan. The post-pandemic period was defined by official reopening declarations and >85.0% population vaccination coverage with two doses.

Campinas, with 1.4 million inhabitants and São Paulo State’s third-largest city, operates a trauma system coordinated by SAMU 192. The trauma system comprises four public hospitals providing 24/7 services: Hospital de Clínicas da Unicamp (HC-UNICAMP), which has 10 dedicated trauma intensive care units and serves as the regional Level 1 trauma center; Hospital Municipal Dr. Mario Gatti, which handles the highest trauma volume; Hospital Ouro Verde, which serves a densely populated region; and Hospital PUC-Campinas. Pre-hospital care is provided, including SAMU 192 and Grupo de Resgate e Atenção às Urgências (GRAU).

Data were collected using databases from the Traffic Engineering Company (Empresa Municipal de Desenvolvimento de Campinas [EMDEC]) and the Traffic Information System (INFOSIGA-SP). All RTIs (fatal and non-fatal) were included. Road traffic deaths were defined as occurring at the scene or within 30 days of the road traffic crash, according to WHO criteria. Data quality was ensured through cross-validation between EMDEC and INFOSIGA-SP databases. Discrepancies were resolved through a manual review of police reports. COVID-19 lockdowns did not significantly impact data collection as both systems maintained electronic reporting throughout the study period.

The primary outcome measure was road traffic mortality. Secondary outcomes included RTI severity and years of potential life lost (YPLL). YPLL was calculated using Brazil’s life expectancy (76.8 years) minus age at death, summed for all premature deaths. Injury Severity Score (ISS)^4^ was calculated using scoring anatomical injuries from 1 (minor) to 6 (maximum) across six body regions, with severe injury defined as ISS >15. ISS data were obtained from hospital medical records and trauma registries. The analyzed variables included road safety indicators (vehicle fleet, average daily traffic volume, and traffic violations), road traffic crash characteristics (vehicles, total crashes, victims, and fatalities), victim profile (demographics and trauma mechanism based on ICD-10 Road Traffic Injuries V01-V59), circumstantial factors (time of day, location, and alcohol testing), and clinical outcomes (ISS, hospital stay, and autopsy findings). Speeding was defined as excessive speed (>60 km/h in urban areas or >100 km/h on highways) or any speed violation recorded by traffic enforcement. Alcohol measurement corresponded to ≥0.05 mg/L in exhaled alveolar air^5^.

Descriptive statistics were used for frequencies and percentages for categorical variables. Chi-square tests were used to compare proportions across periods, with p<0.05 considered significant. Cramer’s V coefficient assessed association strength. The Cochran-Armitage test evaluated trends across periods. Multivariate logistic regression analysis was performed to identify independent risk factors associated with road traffic deaths. Variables were selected for inclusion in the multivariate model if they showed p<0.05 in univariate analysis or were considered clinically relevant based on literature review^6^. The final model included age, gender, speeding, time of day, day of week, location, vehicle type, speeding, and alcohol use. All variables were entered simultaneously using the enter method. Results were presented as odds ratios (ORs) with 95% confidence intervals (CIs). Analyses were performed using SAS System for Windows (Statistical Analysis System), version 9.4. (SAS Institute Inc., 2002-2012, Cary, NC, USA). The study was approved by the Research Ethics Committee of the University of Campinas (CAAE: 65664922.3.0000.5404).

### Data availability statement

The entire dataset supporting the findings of this study is available upon request to the corresponding author.

## RESULTS

**Figure 1.**
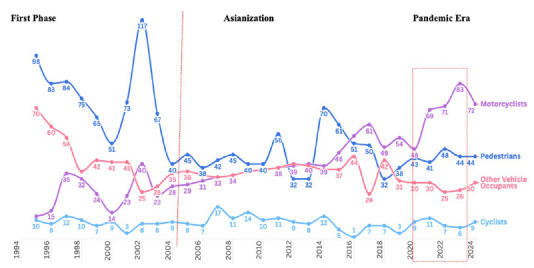
Historical trends in road traffic deaths in Campinas (SP) from 1994 to 2024.

Road safety indicators showed significant changes throughout the study period (Table 1). Despite modest vehicle fleet growth (+2.6%), average daily traffic volume decreased by 32.0% during COVID-19, with partial recovery post-COVID-19 (13.7% below pre-pandemic levels). Paradoxically, mortality rates increased from 10.46 to 13.76 per 100,000 inhabitants, with road traffic deaths becoming more frequent (from one death every 2.9 days to one every 2.0 days) (Table 1). Road traffic mortality rates are demonstrated in Figure 1.

**Table 1.** Traffic safety indicators across pandemic periods.

Indicators	Pre-COVID	COVID	Post-COVID	p-value*
Vehicle fleet	917,120	922,266	947,743	-
Average daily volume (vehicles/month)	81,880	55,677	70,658	<0.0001
Daily traffic violations	1,675	2,126	1,557	<0.0001
Total accidents	4,279	4,257	4,326	-
Total victims	5,786	5,528	6,412	<0.0001
Fatal victims (%)	126 (2.17)	129 (2.38)	151 (2.42)	0.7811
Days between fatalities	2.9	2.4	2.0	-
Mortality rate (per 100,000 inhabitants)	10.46	10.71	13.76	0.5183^†^

*ꭓ^2^ test; ^†^Cochran-Armitage test for trend.

Road traffic crash mechanisms remained stable throughout the study period (p=0.5340), maintaining consistent proportions between collisions, pedestrian strikes, and fixed object impacts.

Analysis of road traffic death patterns (Table 2) showed a progressive increase in nighttime deaths (7 PM-7 AM): 53.1% pre-COVID-19, 62.0% during COVID-19, reaching 64.9% post-COVID-19, with weekend nights accounting for 70.0% of these deaths. Victim demographics remained consistent, with a male predominance (>80.0%) and the highest mortality rate observed in individuals aged 18-29 years.

**Table 2.** Characteristics of fatal victims of road traffic injuries across COVID-19 periods in Campinas (SP), 2019-2023.

Characteristics	Pre-COVID n=126 (%)	COVID n=129 (%)	Post-COVID n=151 (%)	p-value*
Gender				
Male	105 (83.33)	113 (87.60)	133 (88.08)	0.4648
Female	21 (16.67)	16 (12.40)	18 (11.92)	
Age group (years)				
<18	2 (1.59)	10 (7.75)	2 (1.32)	0.0765
18-29	39 (30.95)	33 (25.58)	52 (34.44)	
30-39	29 (23.02)	21 (16.28)	33 (21.85)	
40-49	21 (16.67)	26 (20.16)	31 (20.53)	
50-59	14 (11.11)	19 (14.73)	14 (9.27)	
≥60	21 (16.67)	20 (15.50)	19 (12.58)	
Traffic				
Highway	66 (52.38)	69 (53.49)	75 (49.67)	0.8033
Urban	60 (47.62)	60 (46.51)	76 (50.33)	
Daytime				
Day (7 AM-7 PM)	59 (46.83)	49 (37.98)	53 (35.10)	0.1245
Night (7 PM-7 AM)	67 (53.17)	80 (62.02)	98 (64.90)	
Vehicle type				
Motorcycle	54 (42.86)	50 (38.76)	71 (47.02)	0.3189
Car	31 (24.60)	32 (24.81)	25 (16.56)	
Pedestrian	38 (30.16)	38 (29.46)	48 (31.79)	
Bicycle	3 (2.38)	9 (6.98)	7 (4.64)	
Alcohol testing				
Positive	47 (37.30)	35 (27.13)	62 (41.06)	0.1275
Negative	46 (36.51)	60 (46.51)	51 (33.77)	
Not tested	33 (26.19)	34 (26.36)	38 (25.17)	

*ꭓ^2^ test.

The characteristics of fatal motorcycle injury victims across the three COVID-19 periods are summarized in Table 3.

**Table 3.** Characteristics of fatal victims of motorcycle-related injuries across COVID-19 periods in Campinas (SP), 2019-2023.

Characteristics	Pre-COVID n=54 (%)	COVID n=50 (%)	Post-COVID n=71 (%)	p-value*
Demographics				
Male victims	50 (92.6)	46 (92.0)	67 (94.4)	0.4648
Age 18-29 years	25 (46.3)	22 (44.0)	35 (49.3)	0.0765
Crash characteristics				
Collision with a vehicle	31 (57.4)	28 (56.0)	39 (54.9)	0.5340
Single-vehicle crash	23 (42.6)	22 (44.0)	37 (52.1)	
Safety equipment				
Helmet use	46 (85.2)	43 (86.0)	60 (84.5)	0.8245
Time and location				
Night (7 PM-7 AM)	29 (53.7)	31 (62.0)	46 (64.8)	0.1245
Weekend	38 (70.4)	35 (70.0)	50 (70.4)	0.2459
Highway	23 (42.6)	21 (42.0)	35 (49.3)	0.8033
Risk factors				
Alcohol positive	14 (25.9)	13 (26.0)	23 (32.4)	0.1275
Speeding	11 (20.4)	12 (24.0)	23 (32.4)	0.0828
Survival				
On-scene death	31 (57.4)	29 (58.0)	43 (60.6)	0.3706
Mortality rate				
Per 100,000 inhabitants	4.48	4.15	6.47	0.0328

*ꭓ^2^ test.

YPLL demonstrated substantial societal impact, with young adults (18-29 years) showing a 26.7% increase from pre-pandemic levels.

Trauma severity characteristics across three distinct periods: pre-COVID, COVID, and post-COVID was showed in Table 4.

**Table 4.** Trauma severity analysis across COVID-19 periods.

Characteristics	Pre-COVID (%)	COVID (%)	Post-COVID (%)	p-value*
Injury Severity Score (ISS)				
ISS >25	15	20	17	0.0366
ISS >15 (motorcyclists)	70	70	70	
Hospital stay				
<24 h	22	26	21	0.3706
Autopsy findings				
Traumatic brain injury	65			0.2459
Thoracic vascular injury	15			
Abdominal vascular injury	20			

*ꭓ^2^ test.

Multivariate logistic regression analysis of 17,726 road traffic crash victims (Table 5) revealed that speed-related mortality increased substantially from pre-pandemic to post-pandemic periods (24 vs. 33%). The analysis identified a clear risk hierarchy: speeding combined with alcohol consumption showed the highest mortality risk (OR 2.20; 95%CI 1.49-3.25; p<0.0001), followed by alcohol combined with nighttime driving (OR 2.13; 95%CI 1.44-3.15; p=0.0002), and speeding alone (OR 1.80; 95%CI 1.34-2.42; p<0.0001). Additional significant factors included nighttime speeding (OR 1.65; 95%CI 1.21-2.26; p=0.0015) and highway speeding (OR 1.50; 95%CI 1.07-2.09; p=0.0182).

**Table 5.** Multivariate analysis of 17,726 road traffic crash victims.

Characteristics	Odds ratio (95%CI)*	p-value
Significant risk factors		
Alcohol and nighttime driving	2.13 (1.44-3.15)	0.0002
Nighttime driving (7 PM-7 AM)	1.72 (1.15-2.56)	0.0082
Male gender	1.56 (1.01-2.41)	0.0441
Highway location	1.43 (1.06-1.93)	0.0186
Weekend time	1.38 (1.02-1.87)	0.0366
Age (per year)	1.01 (1.00-1.02)	0.0259
Speeding^†^	1.80 (1.34-2.42)	0.0001
Speeding and highway location	1.50 (1.07-2.09)	0.0182
Speeding and alcohol use	2.20 (1.49-3.25)	0.0001
Speeding and nighttime driving	1.65 (1.21-2.26)	0.0015
Borderline significant factors		
Motorcycle user	1.51 (0.97-2.35)	0.0672
Alcohol use alone	1.39 (0.96-2.01)	0.0828

*Analysis adjusted for age, gender, time of day, day of week, location, vehicle type, and alcohol use; ^†^>60 km/h urban or 100 km/h highway.

## DISCUSSION

RTIs constitute a significant global public health problem, creating multidimensional impacts that transcend immediate healthcare provision. Road traffic deaths impose a considerable burden on healthcare systems across pre-hospital and specialized hospital care, affecting society, mental health, the economy, and the environment. COVID-19 has intensified these challenges as healthcare systems struggled to balance traffic-related trauma care with pandemic response measures. This dual burden highlights the complex relationship between road traffic safety and public health infrastructure, particularly when healthcare resources are redirected toward pandemic control. Both conditions share important characteristics: they produce intergenerational impacts on affected families, generate long-term physical and psychological sequelae in survivors, and impose substantial costs on healthcare and social security systems. The 26.7% increase in YPLL, primarily affecting the productive age group, underscores its substantial societal impact.

One of the most significant pandemic impacts was on urban mobility. Global containment measures resulted in reductions in urban traffic volume during the initial pandemic phases. This reduction was particularly pronounced during lockdowns, as Saladiè et al.^7^ demonstrated in Spain with a 76% reduction in motor vehicle mobility and 93% in public transport. Mobile device geolocation analysis by study with mobile geolocation analysis revelealed^8^ revealed that mobility reductions were geographically and socioeconomically uneven, with higher-income regions and remote work-compatible areas showing greater decreases. De Vos et al.^9^ observed sharp declines in public transport use due to contagion fears, accompanied by increased individual and active transportation modes. While high-income nations reported substantial lockdown reductions, Campinas patterns aligned with countries showing moderate decreases^10^ ^,^ ^11^ ^,^ ^12^: Australia (43.0%), the Netherlands (35.0%), and the USA (40.0%). A striking paradox emerged in our analysis. Traditional traffic safety theory suggests that reduced traffic volume leads to fewer fatalities^10^ ^,^ ^13^ ^,^ ^14^ ^,^ ^15^ ^,^ ^16^. However, our data revealed the contrary: mortality rates increased from 10.46 to 13.76 per 100,000 inhabitants across periods, with increased road traffic deaths during both lockdown phases. This pandemic paradox demonstrates that decreased mobility led to increased mortality rather than improved safety outcomes, challenging conventional assumptions about the relationship between traffic volume and road safety.

Our findings demonstrate a concerning increase in speed-related fatalities during the post-pandemic period, with deaths attributable to excessive speeding rising by 37.5% compared to pre-pandemic levels. This trend aligns with observed traffic pattern changes, where daily violations increased by 27% during reduced traffic periods, with speeding representing 67% of all infractions. Multivariate analysis confirmed speeding as a significant independent predictor of fatal outcomes, with an 80% increased likelihood of death. This finding is particularly concerning, given that speeding violations became more prevalent precisely when overall traffic volume decreased. Nighttime and highway speeding demonstrated amplified mortality risks of 65% and 50%, respectively, likely attributable to reduced visibility during nighttime hours and higher kinetic energy involved in highway crashes.

This behavioral shift aligns with globally documented observations. Katrakazas et al., using sophisticated algorithms, identified a 2.27 km/h average speed increase (6.0-11.0%) in Greece during lockdown periods^17^. Even more dramatic increases were recorded in Spain (39.0%), France (16.0%), the United Kingdom (15.0%), and Denmark (10.0%)^13^. These patterns parallel findings from Virginia, USA, where road traffic deaths increased by 78.0% despite 45.0% fewer crashes^16^.

Pan American Health Organization^18^ demonstrates that speeding contributes to 30% of road traffic deaths in high-income countries and 50% in low- and middle-income nations, reaching 70% in urban crashes involving pedestrians and cyclists. A study^19^ revealed an exponential relationship between absolute speed and crash risk, showing that absolute speed, rather than speed dispersion, constitutes the primary safety determinant. Every 1% increase in average speed results in a 4% increase in death risk and a 3% increase in severe injury^18^. This evidence supports strategies targeting both mean speed reduction and decreased speed variability.

The pandemic’s impact extends beyond traffic patterns, particularly affecting risk behaviors. Other authors^20^ ^,^ ^21^ identified changes in crash patterns, with increases in those related to excessive speeding and substance use. Our findings demonstrated that alcohol involvement in road traffic deaths increased from 27.1% during COVID-19 to 41.0% post-COVID-19, aligning with global trends. Thomas et al.^22^ reported an increase in alcohol consumption among severely injured patients from 51.0% to 65.0% during the pandemic. This was attributed to pandemic-related psychological stress, anxiety, and increased free time during lockdowns, leading to a 14.0% increase in alcohol consumption in Washington, USA^23^. Our analysis demonstrated that alcohol consumption combined with speeding represented the highest risk factor, with 120% increased mortality risk, while alcohol consumption combined with nighttime driving showed the second highest risk. Alcohol use alone approached statistical significance. Our findings identify a lethal triad in traffic safety: alcohol consumption, excessive speeding, and nighttime driving. This synergistic effect dramatically amplifies fatal crash risk when these factors converge.

Similar findings were reported by Alfaro et al.^24^ in Chilean cities, where young males consistently overrepresented both speeding- and alcohol-related deaths. This pattern extends across Latin America, as evidenced in Colombia^25^ where 34% of driver and 23% of motorcyclist deaths were associated with alcohol consumption combined with speeding. Alcohol consumption frequently associates with additional risk behaviors, including non-use of safety devices (seatbelts and helmets), excessive speeding, and concomitant use of other psychoactive substances, amplifying the impairment of psychomotor skills necessary for safe driving.

Risk homeostasis theory^26^ suggests that individuals maintain an intrinsic target risk level, constantly adjusting their behavior accordingly. In traffic contexts, safety improvements are often compensated by riskier behaviors. People accept subjectively estimated risk based on previous experiences, crash potential assessment, and confidence in vehicle control abilities.

This interaction between risk factors creates a particularly hazardous environment, which is further amplified by reduced traffic volume, creating a false sense of security and decreased enforcement during pandemic restrictions. While specific data on traffic agent deployment during lockdowns were not systematically recorded, reports from EMDEC indicated reduced street-level enforcement capacity. This reduction in visible enforcement may have contributed to drivers’ perception. In a paradoxical reconfiguration of mobility dynamics, drivers interpreted low-density traffic streets not as a public health measure, but as an opportunity for excessive speeding, frequently associated with alcohol consumption, thereby significantly amplifying crash risk and severity.

A concerning post-COVID-19 rebound effect emerged in Campinas. Instead of returning to pre-pandemic patterns, mortality rates increased by 31.0%, with road traffic deaths becoming more frequent. Traditional risk behaviors - speeding, alcohol consumption, and traffic violations - intensified during the lockdown and persisted at elevated levels rather than reverting to pre-pandemic baseline.

Global road traffic crash patterns during the COVID-19 pandemic showed significant heterogeneity, with crash reductions varying substantially between countries: from dramatic decreases in France (74.0%) and Spain (67.0%) to moderate reductions in the United Arab Emirates (33.0%), Germany (23.0%), and USA regions (11.0-58.0%)^13^ ^,^ ^14^ ^,^ ^27^. In Campinas, the moderate crash reduction (35.4%) aligned with countries showing moderate mitigation, reflecting three key factors: partial adherence to mobility restrictions, sustained motorcycle-dependent essential worker services, and emerging high-risk behaviors during the pandemic period.

Despite a decrease in total crash volume, road traffic crash severity intensified. Our findings demonstrated that road traffic deaths increased in frequency, with pre-hospital mortality rates rising accordingly. Severe trauma (ISS >15) peaked during COVID-19 at 20.0%, accompanied by increased trauma bay deaths. These severity patterns align with global pandemic observations^7^ ^,^ ^10^ ^,^ ^20^ ^,^ ^27^ ^,^ ^28^ ^,^ ^29^. While mild injuries decreased during lockdowns, severe and fatal injuries remained stable, as reported in the USA and the Netherlands^13^ ^,^ ^20^. Globally, trauma centers have documented an increase in severe cases from 35.0 to 63.0%^30^.

Brazilian federal highways maintained consistent road traffic death patterns from 2019 to 2022 (65,000 crashes and 5,300 deaths annually)^31^. In São Paulo state, its federal highways recorded 887 deaths during this period. Our regional analysis revealed a notable contradiction: while urban areas accounted for most crashes, highways showed higher severity and mortality rates.

This pattern reflects the phenomenon of mixed traffic flow, where five high-speed highways intersect with Campinas urban areas, creating zones where distinct traffic patterns collide. Applying the Haddon Matrix^32^ to these highway-urban interface zones reveals unique risk scenarios where different road systems coexist, amplifying crash severity. In the pre-crash phase, drivers experience cognitive mismatch during abrupt speed transitions, exacerbated by vehicle heterogeneity and ambiguous signaling. During crashes, structural incompatibility between vehicles results in more severe damage. Post-crash, jurisdictional discontinuities and frequently obstructed access routes compromise emergency response efficiency, creating a cascade of factors that elevate both crash occurrence and severity in these transition zones.

Analysis by vehicle type revealed that motorcycles emerged as the predominant factor in mortality (43.1% of deaths), showing a 51.0% higher risk of death. Motorcyclists dominated the severe injuries (50.0% of ISS >15), followed by pedestrians. Autopsy findings showed predominant traumatic brain injuries (65.0%) and major vascular injuries. Mortality rate increased across periods (5%).

**Figure 2.**
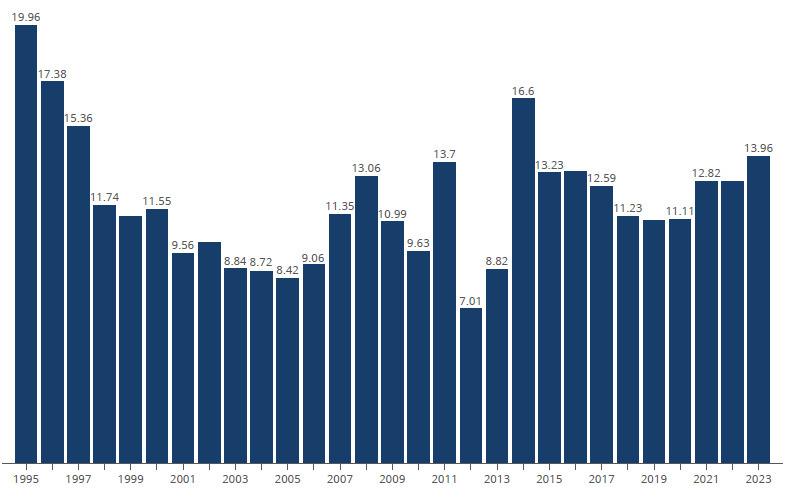
Road traffic mortality rates in Campinas (SP) from 1995 to 2023.

These findings can be explained through a multifaceted analysis. First, Brazil has undergone a paradigmatic transformation in motorization patterns over recent decades - the “Asianization” of traffic through massive motorcycle adoption. This transformation manifested quantitatively through exponential fleet growth: from 1.5 million motorcycles in 1991 to 5 million in 2002, reaching 12 million by 2008^33^. In Campinas, the motorcycle fleet increased by 15.6% from 2019 to 2023. Second, road safety hierarchies demonstrated by Elvik^34^ show that motorcycles present the highest relative crash risk, followed by bicycles, pedestrians, and automobiles, due to factors including limited visibility, vehicle instability, and lack of structural protection. Third, risk behaviors including speeding, traffic violations, and alcohol consumption are amplified during periods of reduced enforcement. Finally, the pandemic-driven expansion of motorcycle-based services, fueled by declining public transport and economic necessities, introduced young and inexperienced riders into motorcycle-based employment during the economic crisis. This vulnerability was particularly pronounced during lockdowns when delivery services intensified and traffic enforcement decreased (Figure 2).

In contrast to high-income countries where motorcycle fatalities decreased, such as Northern Ireland (16.0%) and Australia (12.0%)^13^, this vulnerability during lockdown was not unique to Campinas. Czechia^13^ reported similar increases in vulnerable road user deaths, particularly motorcyclists, suggesting a common phenomenon in regions where two-wheelers remained essential for mobility. Our pattern aligns with broader trends in low- and middle-income countries, where death risks are threefold despite having less than 1.0% of global motor vehicles^1^.

Despite their smaller proportion, bicycle-related deaths showed a threefold increase during the COVID-19 pandemic. In our analysis, cyclist deaths during the pandemic showed a bimodal distribution: half occurred during work-related trips, as active mobility replaced shared public transport to minimize viral transmission risk; the other half occurred during recreational activities, reflecting cycling adoption for mental health preservation during lockdown restrictions. This pattern contrasts with that observed in Australia, where cyclist deaths increased by 29.0% during the lockdown, which appears primarily linked to increased recreational rather than commuter cycling^35^. While Croatia^13^ reported zero pedestrian deaths in April 2020, Campinas maintained its pre-pandemic mortality rates.

Road traffic safety traditionally operates through four fundamental pillars known as the “Four E’s”: engineering, education, enforcement, and emergency response. However, as Tiwari and Mohan^36^ emphasize, conventional approaches focusing solely on compliance through education, training, regulation, and enforcement have proven insufficient to significantly reduce road traffic crash rates, highlighting the need for integrated, multifaceted strategies. Our multivariate analysis identified risk patterns that aligned with the WHO^1^ global framework, showing concerning trends, particularly relevant for low-income countries. The post-COVID-19 mortality rate and death risk increased by 31.0%, indicating that pandemic-related behavioral changes became entrenched rather than temporary.

Our findings support the need for traffic safety governance and management to protect vulnerable road users and prevent traffic deaths. We propose an evidence-based, multifaceted strategy that includes:

1. Strategic nighttime alcohol enforcement checkpoints;
2. Targeted educational programs for high-risk groups, particularly young drivers and motorcyclists;
3. Integrated road safety management involving healthcare professionals, society organizations, traffic engineers, and policymakers; and
4. Reform of motorcycle drive license requirements, particularly for commercial riders.

Professional motorcycle training represents a rational and necessary strategy, given the alarming statistics.

Nevertheless, the pandemic experience offered valuable lessons for urban and traffic planning. A particularly relevant aspect we identified is the need to incorporate public health considerations into urban mobility planning, not only for prevention but also to recognize the broader benefits of active mobility for population health. This underscores the necessity for an integrated approach between public health and road safety sectors.

This study has several limitations. Data collection constraints included the inability to monitor real-time traffic and behavioral metrics. Temporal design issues emerged from seasonal variations and lockdown effects. Analytical limitations stemmed from sample size constraints and reduced statistical power. Behavioral assessments lacked data on the underlying causes of behavioral changes and adaptation patterns.

A pandemic paradox emerged in Campinas, where reduced traffic volumes paradoxically led to increased road traffic mortality, challenging fundamental assumptions about the relationship between traffic density and safety outcomes. Our analysis revealed a lethal triad of traffic risk factors - alcohol consumption, excessive speeding, and nighttime driving - that synergistically amplified fatal crash risk during pandemic restrictions. Young male motorcyclists represented the most vulnerable population, with injury severity significantly exceeding other road users.

Traditional risk behaviors intensified during lockdowns and persisted at elevated levels post-pandemic rather than returning to baseline levels. Our findings not only demonstrate how COVID-19 altered traffic patterns and amplified risk behaviors but also reveal critical opportunities for enhancing road safety interventions through integrated public health.
